# Optimizing 5G NR link layer parameters for eMBB and URLLC applications under dynamic channel and transmission configurations

**DOI:** 10.1038/s41598-025-34674-0

**Published:** 2026-04-02

**Authors:** Sulekha Pateriya, Shuvabrata Bandopadhaya, Amit Kumar Bairwa, Vanshika Jain

**Affiliations:** 1 Department of Physical Sciences, 5G Lab, School of Physical Sciences, Banasthali Vidyapith, Niwai, Rajasthan India; 2 Department of Artificial Intelligence and Machine Learning, School of Computer Science and Engineering, Manipal University Jaipur, Jaipur, Rajasthan India

**Keywords:** Engineering, Mathematics and computing

## Abstract

1000 frames were simulated in MATLAB-based 5G NR link-layer evaluations under 3GPP-compliant conditions to guarantee good statistical reliability. Under realistic propagation conditions, the study concentrated on the following: Downlink shared Channel (DLSCH), Physical Uplink Shared Channel (PUSCH), Physical Uplink Control Channel (PUCCH), Physical Downlink Shared Channel (PDSCH) and Hybrid Automatic Repeat Request (HARQ). The impacts of multipath delay spread, Doppler shifts, and user mobility were captured using standardized channel models, such as CDL-A to CDL-D and TDL-B100. The study looked at dynamically changing transmission parameters, such as frequency-hopping strategies, subcarrier spacings between 15 and 120 kHz, and modulation schemes ranging from QPSK to 256-QAM. The findings showed that while larger subcarrier spacings (60–120 kHz) improved throughput in high-SNR and low-latency scenarios, smaller subcarrier spacings (15–30 kHz) provided better block error rate (BLER) performance in low-SNR and high-delay conditions. Moreover, QPSK proved resilient in noisy settings, whereas 256-QAM reached maximum throughput in favourable SNR conditions. Interestingly, PUCCH with interest frequency hopping had the lowest BLER, demonstrating that it works well in channels that are dominated by fading. The results highlight how important adaptive link-layer configurations are for optimizing spectral efficiency and guaranteeing dependable performance in a range of deployment circumstances. To meet the demanding needs of ultra-reliable low-latency communication (URLLC) and enhanced mobile broadband (eMBB) services in next-generation wireless networks, these insights are essential.

## Introduction

The widespread adoption of fifth-generation 5G New Radio (5G NR) has been driven by the increasing demands of mobile broadband and mission-critical communication services. These networks have been characterized by a flexible physical layer design alongside a highly adaptive medium access control (MAC) layer. Foundational elements of the 5G NR air interface, including the Physical Downlink Shared Channel (PDSCH), Physical Uplink Shared Channel (PUSCH), Physical Uplink Control Channel (PUCCH), and the Hybrid Automatic Repeat Request (HARQ) are defined as integral to the link layer. These components are required to ensure the reliability, efficiency, and adaptability of data and control transmissions across diverse operational scenarios^1–3^.

In 5G NR, PUCCH transports uplink control information such as HARQ feedback, scheduling requests, and Channel State Information (CSI), while PDSCH and PUSCH transport downlink and uplink data transmission, respectively. HARQ provides reliability through the use of a combination of Forward Error Correction (FEC) and acknowledgment signaling. Uplink user data travels through the Uplink Shared Channel (UL-SCH) at the logical layer before being transferred to the PUSCH for transmission over the air. According to 3GPP (3rd Generation Partnership Project), while PUCCH utilizes short block or repetition codes to provide reliable performance across a wide range of radio conditions, PDSCH and PUSCH employ Low-Density Parity-Check (LDPC) coding. It has been necessary for these link-layer methods to function in dynamic and unpredictable environments, such as fluctuating interference, Doppler dispersion, user mobility, and frequency-selective fading. Although 3GPP TS 38.901 specifies extensive channel models for indoor, vehicle, rural, and urban settings^4^. Many studies have been found to employ static and simplistic configurations. Consequently, there is currently a lack of thorough investigation into the realistic operating performance of these link-layer processes in dynamic situations. Performance analysis of PDSCH, PUSCH, and PUCCH under perfect scheduling or channel conditions have been performed in earlier studies that focus on certain deployment environments^5–7^. Optimal performance or downlink physical process modeling under certain conditions has been achieved in some research^8^. MATLAB-based simulation platforms have facilitated easier waveform development^9^, whereas real-time emulation remains challenging. Efforts such as Inclusive Radio Communication Networks for 5G and Beyond (IRACON), which outlined relevant propagation characteristics over frequencies and environments, has significantly improved channel modelling^10^.

Yet, research gaps were critical. Specifically, the absence of dynamic testing frameworks has kept full evaluation of link-layer behaviors under different traffic demands, CSI variations, and user mobility^11^,and^12^ in check. Furthermore, enough quantitative information on the effectiveness of PUCCH format selection, DMRS bundling, and frequency hopping between slots still falls short of adequate quantification for deployment realities^13^. While all these techniques are anticipated to improve link reliability, particularly for cell-edge users, very little is known about how they would behave in varying HARQ, modulation, and coding, and dynamic Subcarrier Spacing (SCS) configurations. In this study, we fill these gaps by assessing the performance of 5G NR systems in actual scenarios through simulations spanning 1000 frames, which is a significant improvement over previous research that only examined 2–10 frames^14–16^. We can record a wider range of channel fluctuations, interference patterns, and error behaviors thanks to this increased frame count, which yields more accurate and statistically meaningful results. To examine how various signal-to-noise ratio (SNR) situations affect throughput and Block Error Rate (BLER), we apply channel coding, MIMO modulation, and multiple subcarrier spacings.

To identify configurations that optimize performance in both low-SNR and high-SNR scenarios, we will investigate a wide range of parameters, including modulation techniques up to 256-QAM, bandwidth variations, and realistic urban macro cell channel models. By providing greater insights into the dynamic behavior of 5G systems over long transmission periods, our work improves the accuracy of network design and optimization tactics, advancing the state of the art. It is proposed in this work to address these concerns with a simulation-based investigation of PDSCH, PUSCH, PUCCH, and HARQ performance under various channel and transmission parameters. The following contributions have been compiled:

1. A thorough analysis of PDSCH, PUSCH, and PUCCH under different subcarrier spacings (15–120 kHz) and modulation formats (QPSK, 16QAM, 64QAM, and 256QAM), considering coding and adaptation techniques provided by 3GPP^1,17,18^.
2. To ensure realistic and completely repeatable simulation conditions, performance evaluations were conducted using comprehensive 3GPP TR 38.901–compliant propagation models that included Doppler shift, path loss, and multipath fading based on CDL and TDL channel profiles (e.g., CDL-B with a 300-ns delay spread), along with their specified LOS/NLOS characteristics^12^.
3. Quantitative analysis of the control-plane signaling robustness, throughput, and BLER under various scheduling and mobility conditions, e.g., varying interference, frequency hopping, and HARQ configurations^4,13^.
4. For optimizing link-layer architecture to accommodate greater mobile broadband (eMBB) and ultra-reliable low-latency communications (URLLC) services, an analytical comparison of spectral efficiency, data-plane reliability, and control-plane robustness trade-offs were conducted^2,19,4^.

The remainder of this paper is organized as follows. The research problem is presented, and its importance is explained in Sect. “Introduction”. A literature review that identifies the current research gap is included in Sect. “Background and theoretical framework”, together with the theoretical framework and background information, coupled with mathematical formulations to support it. In Sect. “Methodology framework description”, the research methodology is explained, including the methods and strategies used in the suggested work. Results and analysis are presented in Sect. “Results and discussion”, and the findings are discussed in Sect. “Discussion”, with a focus on the contribution of adaptive link configuration to enhancing 5G NR performance in a variety of deployment scenarios. Section “Conclusion and future work” wraps up the work by providing a summary of the main contributions, outlining potential model improvements, and suggesting future research avenues.

## Background and theoretical framework

A key component in optimizing the performance of 5G NR systems is the relationship between throughput and SNR. Channel Models, SCS, and modulation techniques are important affecting factors. Better-order modulation techniques, such as 256-QAM, are more susceptible to impairments like noise and inter-symbol interference (ISI), but they can achieve noticeably better throughput in high-SNR situations.QPSK, on the other hand, offers resilience in low-SNR situations, but at the expense of a lower spectral efficiency^12,20^.

In order to balance latency, spectral efficiency, and throughput, subcarrier spacing is essential. In channels with limited coherence, boosting SCS can worsen performance, but it can also use frequency diversity to increase throughput, particularly When the Signal bandwidth surpasses the channel coherence bandwidth. Understanding how multipath propagation affects performance requires an understanding of channel modeling, especially Clustered Delay Line (CDL) and Tapped Delay Line (TDL) models^17^.

Additionally, by increasing spatial diversity and permitting retransmissions for increased reliability, link-layer technology like HARQ and antenna designs like massive MIMO have a considerable impact on the throughput–SNR relationship^17^. Therefore, maintaining an ideal trade-off between throughput and BLER requires adaptive parameter selection based on real-time network conditions^12^.

### Literature review

#### Dependency on SNR and modulation schemes

The trade-offs between modulation order, throughput, and BLER have been the subject of numerous studies. Despite sacrificing spectral efficiency, QPSK provides strong performance in low-SNR areas with low BER^17^. A balanced approach is provided by 16-QAM and 64-QAM, with 16-QAM outperforming 64-QAM and 256-QAM in some SCS setups, such as 120 kHz^12^. 256-QAM attains the maximum throughput in high-SNR settings, but its performance deteriorates when channel impairments Are present, as shown in Table 1.

#### Throughput and SCS

By maximizing spectrum utilization and taking advantage of frequency diversity, an increase in SCS can boost throughput, especially when the bandwidth exceeds the coherence bandwidth^17^. However, in situations with poor coherence bandwidth or strong Doppler dispersion, higher SCS values could lead to a loss of performance^12^.

#### Comparing CDL and TDL in channel modeling

The realistic clustered scattering profiles of CDL models make them ideal for assessing the impact of multipath propagation. These models show how complicated multipath effects interact with various modulation and SCS combinations. For channel evaluation, TDL models offer different viewpoints, especially in certain deployment scenarios^20^.

To examine channel behavior under non-homogeneous propagation conditions, more generalized fading distributions have also been added in recent works. For example, the double $\lambda$–$\kappa$–$\mu$ distribution discussed in^21^ expands this framework to cascaded channels, allowing robust evaluation under highly dynamic and non-stationary environments, while the $\lambda$–$\kappa$–$\mu$ distribution presented in improves statistical modeling accuracy for LOS-dominant multipath scenarios. These theoretical distributions offer complementary insights that support the importance of thorough fading characterization for sophisticated 5G NR performance analysis, even if they are not directly incorporated in 3GPP-based simulations^22^.

#### Mechanisms at the link layer

1. HARQ: When properly configured, it improves throughput, lowers BLER in unfavorable channel circumstances, and retransmits incorrect packets to increase dependability^17,23^.
2. PDSCH/PUSCH: Although the best setups for up-link and downlink are different, throughput increases with modulation order and SCS^17,20^. The BLER sensitivity of PUCCH, which is mostly utilized for control signaling, affects network stability as opposed to data throughput^1,24^. However, without specifically evaluating PUCCH configuration under SCS and modulation modification, the majority of current research^25,26^ focuses on general NR performance. Similar to this, whereas research like^27^ look at HARQ, the combined effect of uplink control channel performance is still not well studied; this work fills that gap.

#### Configurations of antennas

Through array gains and spatial diversity, massive MIMO and higher antenna counts enhance link stability and throughput even at lower SNR^17^.

**Table 1:** Research gaps in the performance characterization and error-rate analysis of 5G NR link-layer mechanisms.

Author(s)	Year	Findings	Future research
Lauri Sormunen, et al.^28^	2025	While DVB-S2X RCS2 can be more specific, truly efficient, 5G NR NTN offers reduced latency. System configurations and traffic load affect performance.	Test in MEO/LEO scenarios. Add mobility and interference effects. Explore hybrid DVB-S2X/5G NR NTN designs.
Mostafa Rahmani et al.^29^	2024	Reliability and spatial variety are improved more RUs. The performance of frequency diversity is enhanced by higher SCS values.	PDSCH, PUCCH, and PUSCH 5G NR channels should be extended. Add beamforming, coding effects, and MIMO. Apply to THz and mmWave bands. Employ ML-based AMC to reduce BER. Examine BER in hybrid satellite-terrestrial networks and NTN.
Johny Helder Da Silva Dias Pires^30^	2023	The range of 2.1599937 GHz to 2.1600014 GHz was found to have the highest throughput and smallest BLER. A 5G base station can communicate with two 5G NR devices.	Examine uplink cohabitation amongst several 5G NR devices. For low BLER and excellent throughput, choose the ideal frequency range. Examines scaling to connect a large number of devices.
Dharmender Kumar^31^	2023	Allocating resources effectively is essential for 5G network performance. Latency, energy efficiency, and system performance measurements are examples of KPIs.	Enhanced methods for allocating resources in 5G networks tackling frequent and technical 5G issues.
Deepak Sinwar^7^	2023	PDSCH throughput fluctuates according to modulation methods and numerology. On 120 kHz SCS, 16-QAM performed better than 64- and 256-QAM.	No specific future work is mentioned. With experimental data, the study focuses on PDSCH throughput vs. SNR; no recommendations are made for future research topics.
Vladimir Lapin et al.^32^	2022	Evaluates 5G NR PUCCH’s error rate performance and determines the critical factors influencing PUCCH error rates.	No specific future work was specified. Focuses on Format 3 simulation and 5G NR PUCCH error rate analysis to determine important parameters.
Krasen Kirov Angelov et al.^8^	2022	Throughput, BLER, and BER performance analyses were performed. HARQ mechanisms and CQI levels affect the results.	No specific future work was mentioned. 5G NR a downlink PHY simulation model for BER, BLER, and throughput study under varied situations is presented in this paper using MATLAB.
Sutharshun Varatharaajan et al.^33^	2022	Multi-TRP schemes enhance PDCCH reliability and robustness. Effective in improving performance under various channel conditions	No explicit section, but suggests optimizing PDCCH schemes for reduced-capability UEs. Potential to integrate enhancements into flexible network scheduling for enhanced performance.
Yasin Kabalci et al.^5^	2022	In areas with poor SNR, QPSK performs better. 256-QAM performs well in areas with strong SNR.	No specific future work was mentioned. According to the study, 256-QAM performs best at high SNR and QPSK at low SNR; throughput is enhanced by more BS antennas and increased by greater SCS.
Peng Hao et al.^34^	2020	Models of delay and reliability for URLLC traffic are shown. Link-level simulation was used to evaluate the PUCCH resource selection scheme.	Focuses on models, analysis, simulations, and suggested structure; no specific future work is specified.

### Theoretical framework

#### Synopsis of 5G NR link layer mechanisms

According to the 3GPP, the 5G NR air interface is specified to provide a scalable and adaptable framework capable of supporting a diverse range of service types and deployment scenarios^1,2,17^. The link layer, which serves as the foundation of this architecture, is composed of essential elements such as the HARQ, the PUSCH, and the PDSCH.

The transmission of user data in the downlink and uplink directions is facilitated by the PDSCH and PUSCH, respectively. Downlink data is delivered from the gNB to the User Equipment (UE) through the PDSCH, whereas uplink user traffic is transmitted from the UE to the gNB via the PUSCH. Dynamic scheduling and management of these shared channels are accomplished through control signaling transmitted over the Physical Downlink Control Channel (PDCCH)^11^. On top of these channels, the HARQ protocol is employed, where forward error correction (FEC) is integrated with retransmission mechanisms to ensure resilient delivery, there by significantly enhancing throughput and reliability^4,24^.

#### Downlink transmission chain

A variety of modular physical-layer functionalities, which transform data into orthogonal frequency division multiplexing (OFDM) Waveforms, have been employed to construct the down-link transmission procedure in 5G NR.

#### Channel coding and modulation

LDPC coding and rate matching for the PUSCH are also applied according to the same downlink baseline requirements.The MCS configuration determines the M-QAM constellation used in modulation^35^. After mapping the output to a subset of OFDM subcarriers, an inverse fast Fourier transform (IFFT) is applied to convert the signal to the time domain^36^.

#### Reference signals and transmission

Demodulation Reference Signals (DMRS) are multiplexed with PUSCH data for channel estimation purposes. The use of one or more antennas in transmission varies based on the UL MIMO configuration^13^.

#### Uplink control signaling with PUCCH

PUCCH has been assigned to transport UCI to the gNB, including HARQ acknowledgments, CSI feedback, and UE scheduling requests. To support different payload sizes, latency targets, and coverage requirements, 3GPP TS 38.213 specifies several PUCCH formats, including short and long formats. PUCCH transmission is based on uplink reference signals for reliable detection and dynamic scheduling to share uplink resources with PUSCH and achieve low overhead^4,6^. PUCCH formats are also minimized for low-power UE operation using power control and repetition coding schemes. Soft-combining has been utilized to realize HARQ at the receiver. In the event of failure of decoding, retransmission is done utilizing other Redundancy Versions (RVs). The following equation is employed to calculate the effective SNR after retransmissions.

**Fig. 1:**
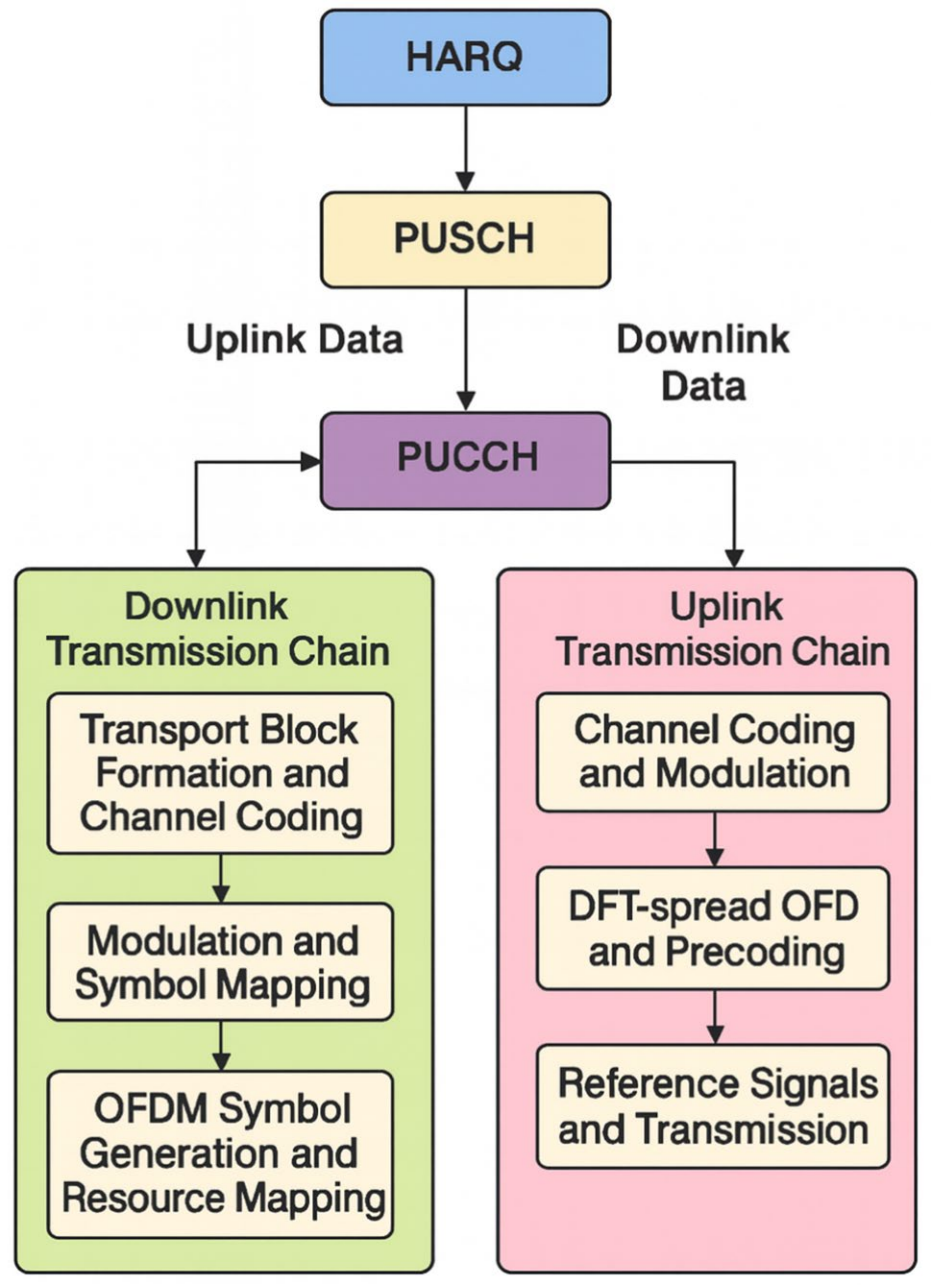
Link-layer mechanism framework in 5G NR: down-link scheduling with HARQ processes and up-link transmission^2^.

Show Fig. 1 in 5G NR, uplink transmission uses grant-based and grant-free techniques for effective access, while the link-layer mechanism framework combines downlink scheduling with HARQ procedures to provide dependable data delivery. When combined, these strategies improve throughput, dependability, and flexibility in a range of network scenarios.

## Methodology framework description

### Theoretical structure

The process starts with the creation of input data and moves methodically through channel simulation, encoding, modulation, and output assessment. The system architecture for PDSCH and PUCCH throughput and BLER simulation is described by the framework using a block-structured flow. Important Modules and Their Relationships.

**Fig. 2:**
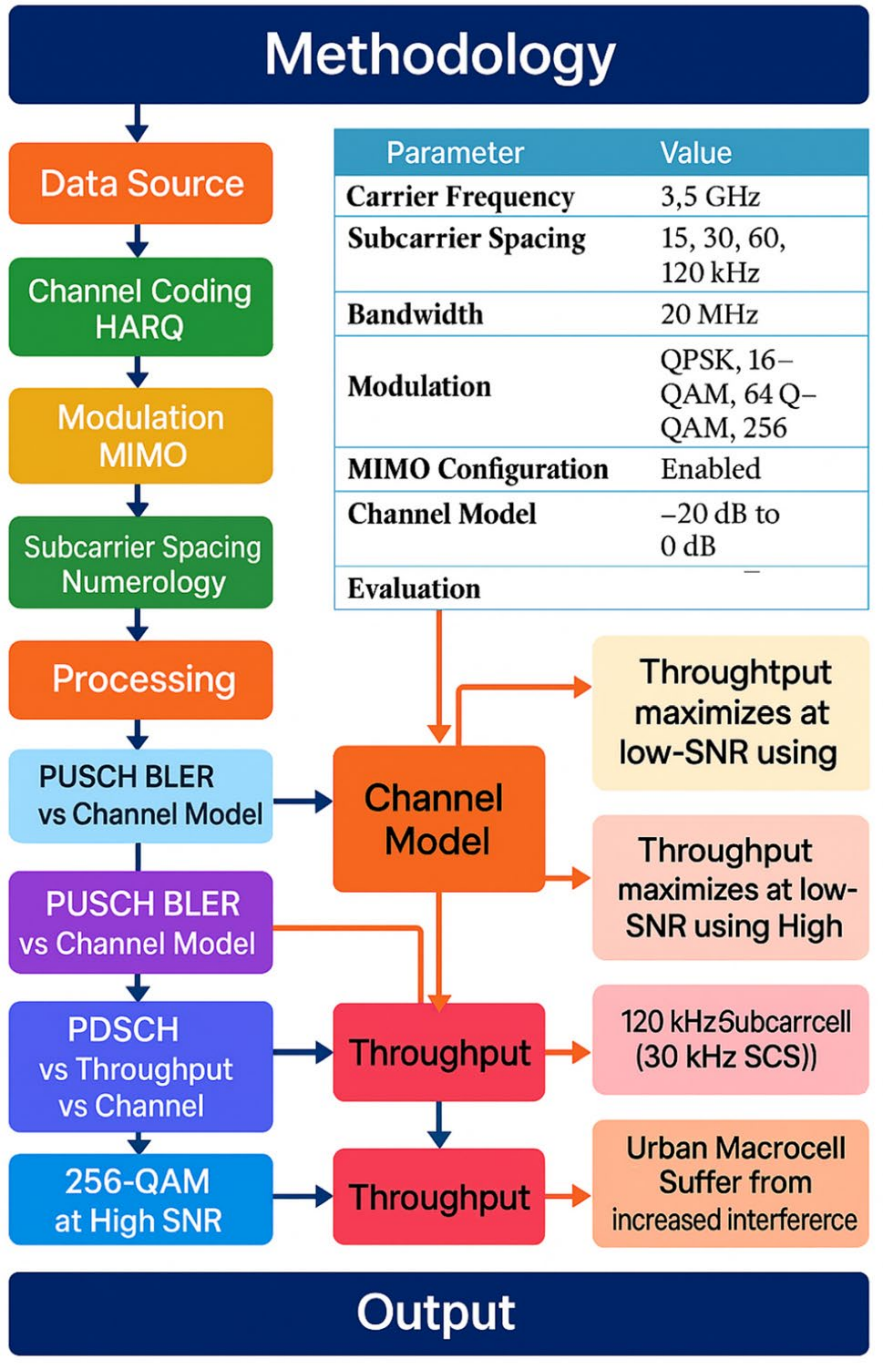
Methodology framework for evaluating PUSCH and PDSCH performance under different 5G NR setups, emphasizing throughput optimization and channel model-based BLER assessment.

Figure 2 shows the methodology approach used to assess 5G NR uplink transmission performance in terms of throughput and Block Error Rate (BLER). Starting from the Data Source, the procedure moves through stages of system configuration, such as MIMO-based modulation, channel coding using HARQ, and numerology selection. The accompanying table provides a summary of the simulation settings, including carrier frequency, subcarrier spacing, bandwidth, modulation techniques, and MIMO setup. The Processing block carries out performance evaluation activities like PUSCH BLER vs. channel model, PDSCH throughput analysis, and high-SNR throughput testing using 256-QAM.

It is noteworthy that in Fig. 2, the arrow that emerges from the “PUSCH BLER vs Channel Model” block points in the direction of the “Channel Model” block, signifying that BLER analysis is carried out under the particular channel characteristics (such as SNR range and propagation parameters) specified by the chosen model. The process is then completed by transferring performance insights to the Throughput block and aggregating them in the Output block.

### Input module

- A user-specified Data Source is loaded by the system.
- For redundancy and dependability, it uses HARQ and Channel Coding.
- For spectral efficiency, it carries out modulation and multiple input multiple output (MIMO) mapping.
- It sets up Numerology and Subcarrier Spacing in accordance with 5G NR requirements.

### Module for processing

- The PDSCH/PUCCH physical layer processing chain, which is 3GPP-standard, is used by the simulation framework.
- The modulated symbols are sent into the transmission buffer by the system.

### Simulated channel

The framework injects the transmission stream into a customizable Simulated Channel, configured for SNR, delay spread, and fading models (e.g., TDL, CDL).

### Output module

The signal is decoded and calculated by the receiver. Metrics of Performance: Throughput of the Physical Layer (bps) BLER.

### Framework for workflow

The simulation runs continuously.

- Create input transport blocks first.
- Utilize PHY-layer setups.
- Process data via mapping, modulation, and HARQ.
- Use TDL/CDL channel models for simulation.
- Decode and demodulate the symbols that were received.
- Compare BLER and throughput to theoretical bounds.

### MATLAB-based simulations

This work uses comprehensive MATLAB-based simulations to assess the behavior of the two most important physical channels of the 5G NR system: PUSCH and PDSCH.The main goal is to investigate how different system parameters affect throughput and Block Error Rate (BLER) in different SNR scenarios, as in Fig. 2.

#### UL-SCH PUSCH (Up-link Shared Channel-Physical Up-link Shared Channel

**Fig. 3:**
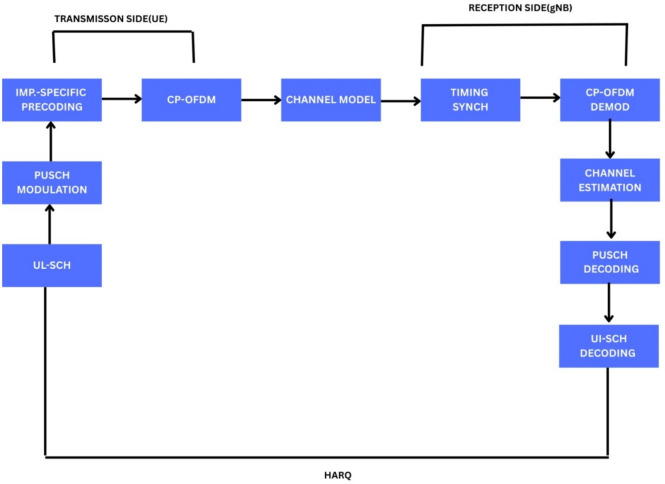
Block-structured uplink PUSCH workflow with HARQ mechanism to improve reliability^37^.

As shown in Fig. 3, the 3GPP TS 38.211, 38.212, and 38.214 simulations have been utilized to evaluate the 5G NR Physical Uplink Shared Channel (PUSCH) performance. Throughput and BLER are the primary topics of study, which consider various physical-layer aspects such as channel models, modulation techniques, and SNR^20^.UL-SCH processing, LDPC coding, and soft Buffer HARQ management with four simultaneous operations is all implemented in the UE transmission.Subcarrier spacing of 15–120 kHz and modulation schemes of QPSK through 256-QAM are utilized, and PUSCH is formed with CP-OFDM. Beamforming is simulated in non-codebook mode under single-user MIMO. Ideal synchronization is presumed at the gNB,and channel models(CDL-A/B/C/D) are utilized. The impact of modulation and channel fluctuation on uplink reliability and spectral efficiency is illustrated through simulations carried out within an SNR of − 10 to +10 dB^12^.

#### DLSCH PDSCH (Down-link Shared Channel - Physical Down-link Shared Channel)

**Fig. 4:**
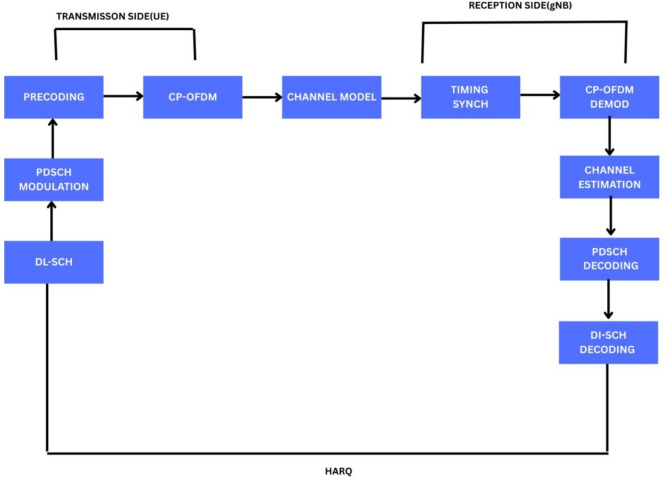
Block-Level Architecture of 5G NR Downlink PDSCH: Transmission Chain with HARQ Operation^37^.

Statistical assessment of 5G NR downlink performance was performed using a MATLAB simulation platform that is like the conventional PDSCH processing chain As show in Fig. 4, Throughput and BLER were examined for several modulation methods, subcarrier spacings, and channel models (CDL and TDL types); HARQ was used to increase reliability^20^. For − 10 to +10 dB SNRs, 2 × 2 and 4 × 4 MIMO topologies were utilized to model the 3.5 GHz frequency band. LDPC coding was used to correct errors forward, and conventional 3GPP resource maps were used.Channel estimation, demodulation, and synchronization were all receiver processing. The following were the findings, which are the trade-offs in robustness and spectral efficiency that led to the physical layer parameters being optimized in realistic 5G propagation environments^1,17,24^.

#### UL-SCH PUCCH (Up-link Shared Channel - Physical Up-link Control Channel)

**Fig. 5:**
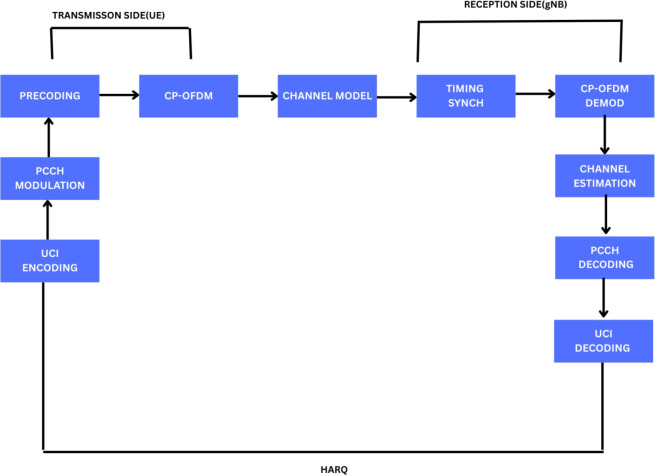
Block-level uplink PUCCH transmission architecture: HARQ-aided dependability in 5G NR^38^.

The 5G NR PUCCH performances has been analyzed using MATLAB simulations, in which UCI reliability has been studied under different SNR scenarios.A 1 × 2 antenna configuration with a carrier frequency of 3.5 GHz and subcarrier spacing of 15 or 30 kHz was used with a TDL fading channel model. As show Fig. 5, BLER was observed over SNR from 20 to 10 dB to study control channel robustness. HARQ controlled the retransmissions to provide uplink control signaling reliability.

### Simulation parameters

To close these gaps, MATLAB-based simulations covering 1000 frames were used to evaluate the performance of 5G NR systems in realistic circumstances, which is a major improvement above earlier research that only looked at 2 to 10 frames 3GPP TR 38.901 criteria are followed in the simulation setup. To ensure consistency and transparency, Table 2 provides a comprehensive description of the channel-related characteristics, such as mobility conditions, Doppler shift, Rician K-factor, and delay spread.

MATLAB R2025a with 5G Toolbox was used for all simulations Resource allocation was done using a conventional Round-Robin scheduling technique and HARQ Type-2 were set up with four retransmission processes and timing in accordance with 3GPP TS 38.321 criteria.

**Table 2:** An overview of the main simulation parameters used to assess the performance of 5G NR links. Every parameter complies with 3GPP TR 38.901 requirements, guaranteeing accurate and repeatable channel modelling circumstances.

Parameter	Value
Carrier Frequency	3.5 GHz
Bandwidth	20 MHz
Subcarrier Spacing (SCS)	15, 30, 60, 120 kHz
Modulation Schemes	QPSK, 16-QAM, 64-QAM, 256-QAM
MIMO Configuration	8 × 2 (8 Tx, 2 Rx)
Channel Model	CDL-B/TDL (as per 3GPP TR 38.901, including LOS/NLOS characteristics)
Delay Spread	300 ns (for CDL-B)
Propagation Conditions	Includes Doppler shift, path loss, and fading as per CDL/TDL profiles
LOS / NLOS Characteristics	CDL-B (Urban macrocell, LOS-dominant), TDL-B/C for NLOS scenarios
Doppler Shift	5 Hz (low-mobility scenario)
Channel Estimation	Perfect channel estimation
Energy per SNR Range	–10 to +10 dB
HARQ Configuration	Enabled (4 processes, RV = [0, 2, 3, 1])
LDPC Decoding Algorithm	Normalized min-sum, 6 iterations
Antenna Array Geometry	Conforming to 3GPP array model (per CDL profile)
Repetitions	5000 channel realizations per SNR point
Frames per SNR Point	1000 frames (convergence validated)
Numerology Mapping	Full-grid allocation (51 PRBs)

### Simulation validation strategy, reliability, and applicability limits

The following formal validation and reliability assessment was carried out to guarantee methodological transparency and boost trust in the generality of the results presented.

#### Model validation

Every simulation result was cross-checked against the standard-compliant behaviour described in 3GPP TR 38.901 and TR 38.212. Consistency with anticipated NR link-level trends was confirmed by the BLER transition threshold points and throughput saturation levels, which closely matched those shown in reference test scenarios and MATLAB 5G Toolbox examples. The reference CDL-B and TDL model requirements were used to validate the channel profile characteristics (RMS delay spread, Doppler shift, and LOS/NLOS conditions).

#### Statistical reliability

One thousand independent frames per SNR point were used in a Monte Carlo study. Statistical stability was demonstrated by the frame convergence examination (extended up to 2000 frames), which revealed a < 0.5% change in findings beyond 1000 frames. Only once dynamic throughput and BLER convergence have been verified are the final findings published.

#### Reproducibility assurance

At every SNR iteration, the random number generator was reset using rng(‘default’) to promote reproducibility. Full replication is made possible by Table 2, which summarizes all simulation settings, including modulation schemes, MIMO configuration, HARQ setup, channel profiles, and decoder configuration. Furthermore, the extra appendix has a list of all 3GPP Toolbox functions that were used, such as nrCDLChannel, nrPDSCHConfig, and nrDLSCHDecoder.

#### Applicability limits

- Perfect channel estimation and synchronization, which may differ from real-world deployments in high-mobility or interference-dense scenarios.
- Low mobility environment (5 Hz Doppler shift, < 3 km/h).
- Urban macrocell conditions represented using CDL-B and TDL-B/E fading models.
- Single-cell link performance (no inter-cell interference).
- Power control and adaptive scheduling across cells are not considered in this model.
- Massive MIMO beamforming is not included and will be incorporated in future extensions of the simulation model.

## Results and discussion

### BLER vs channel models

**Fig. 6:**
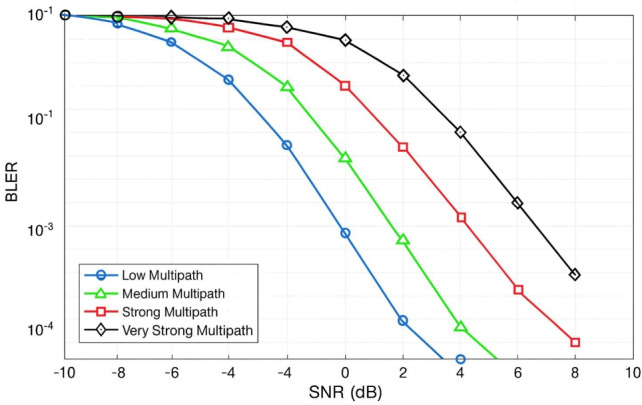
Channel models vs. BLER: Uplink PUSCH performance in 5G NR under TDL and CDL fading scenarios.

As indicated in Fig. 6, BLER is shown for low, moderate, strong, and severe the multipath. At a dismal 2 dB SNR, BLER is still below 10^−2^. As the multi-path deteriorates, it takes more SNR to perform: 4 dB for moderate, 6 dB for severe, and over 7 dB for very severe. Better performance in low multipath environments again underscores its importance in offering the best error performance in lower SNR. These results highlight the necessity of using multi-path reduction techniques and accurate channel estimations, especially in high-delay-spread systems such as indoor and urban wireless systems.

### BLER vs SCS

**Fig. 7:**
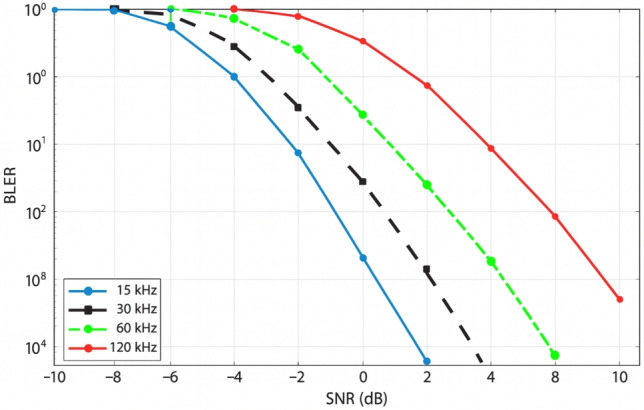
Uplink PUSCH performance in 5G NR: BLER vs. subcarrier spacing (SCS).

Four numerologies with subcarrier spacings of 15 kHz, 30 kHz, 60 kHz, and 120 kHz are taken into account in the simulation. According to the results, BLER performance consistently deteriorates when the SCS rises, especially when multipath delay spread is significant. With a BLER of $10^{-2}$ at an SNR of about 3 dB, the narrower 15 kHz SCS performs the best. On the other hand, because of its shorter symbol duration and greater vulnerability to multipath-induced inter-symbol interference (ISI),The 120 kHz design needs more than 7 dB SNR to reach the same BLER threshold. The performance of the intermediate numerologies, 30 kHz and 60 kHz, likewise deteriorates in proportion to their SCS values. This suggests that lower-SCS numerologies (15 kHz and 30 kHz) are better suited for obtaining dependable data transmission in multipath-rich situations such as urban macro or indoor installations. In low-delay-spread or line-of-sight small-cell circumstances, where latency is more important than robustness,higher SCS values like 60 kHz and 120 kHz are still beneficial. In order to ensure that Doppler effects are minimal, it is crucial to highlight that the simulations in Fig. 7 use a low-mobility scenario ($\approx$ 3 km/h) .According to this theory, multipath-induced ISI alone–rather than Doppler-induced inter-carrier interference—is what causes the observed BLER changes. Wider SCS values ($\ge$ 30 kHz) often offer better robustness than 15 kHz because of their shorter OFDM symbol time, but in higher mobility situations, the Doppler spread becomes a dominating hindrance. The high-mobility results shown later in the text analyze this mobility-dependent behavior.

### Throughput vs channel models

**Fig. 8:**
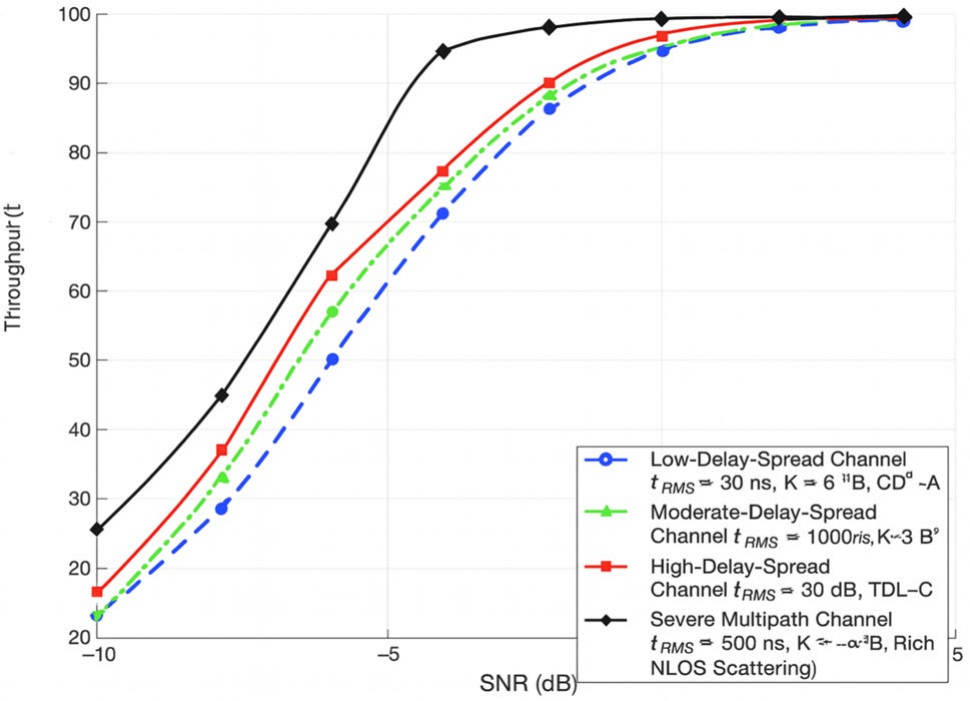
Channel Models vs. Throughput: Uplink PUSCH Performance in 5G NR under TDL and CDL Fading Scenarios.

Instead of using qualitative terminology to describe the multipath circumstances, Fig. 8 uses quantitative 3GPP TR 38.901 characteristics. The scattering richness, LOS/NLOS characteristics, Rician K-factor, and RMS delay spread $(\tau ^{\textrm{RMS}})$ of the channel environments vary considerably. With a short delay spread of about 30 ns and a significant LOS component $(K \approx 6\,\textrm{dB})$ ,the Low-Delay-Spread Channel is an example of CDL-A-type propagation with limited frequency selectivity. The Moderate-Delay-Spread Channel, which corresponds to CDL-B, has a partial LOS component $K \approx 3\,\textrm{dB}$ and a delay spread of about 100 ns. Larger delay spreads of about 300 ns and NLOS propagation (K = 0 dB) result in deeper fading and more noticeable frequency selectivity in the High-Delay-Spread Channel, which is modelled after TDL-C. Lastly, the Severe Multipath Channel represents extreme TDL-E conditions where multipath scattering predominates entirely, the Rician K-factor approaches $-\infty$ dB, and $\tau ^{\textrm{RMS}}$ surpasses 500 ns.

### Throughput vs SCS

For subcarrier spacings (SCS) of 15, 30, 60, and 120 kHz, the throughput performance as a function of SNR (evaluated over the range of $-10$ to 20 dB) is examined. Owing to its longer symbol duration, improved noise resilience, and increased tolerance to frequency-selective fading, the 15 kHz SCS achieves the highest throughput in the low-SNR region. Conversely, the 120 kHz SCS exhibits the lowest throughput at low SNR values due to its limited time-domain averaging and shorter symbol duration, making it more susceptible to noise.

Although larger SCS values are less robust under noisy channel conditions, they are advantageous for high-mobility, low-latency, and mmWave applications, where shorter symbol durations and wider bandwidth prove beneficial. Figure 8 demonstrates that all SCS configurations converge to nearly 100% throughput at sufficiently high SNR levels (approximately 6–8 dB), indicating that the influence of SCS diminishes once the signal strength is sufficiently high. These results highlight the importance of adaptive SCS selection for optimizing system performance across varying channel conditions and deployment scenarios.

###  Downlink PDSCH performance

#### Throughput vs SCS

**Fig. 9:**
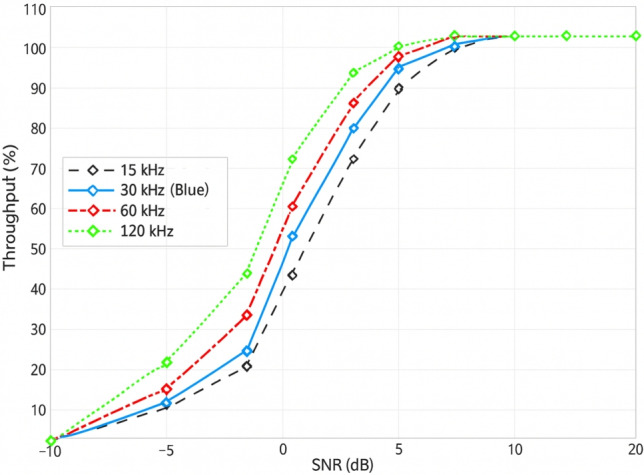
Assessment of PUSCH Throughput with SCS Values of 15 kHz, 30 kHz, 60 kHz, and 120 kHz under TDL/CDL Fading Models in 5G NR.

In the 5G NR system, the plot shows Fig. 9 throughput vs. SNR for various subcarrier spacings (SCS): 15, 30, 60, and 120 kHz. In all the levels of SCS, throughput depends upon SNR, but the rate and efficiency are completely different. The 120 kHz SCS is the best and ideal for high-mobility, high-frequency applications such as URLLC and mmWave. At 10 dB SNR, it is nearly 100% throughput. Most applicable to urban and medium mobility channels, 60 kHz and 30 kHz SCSs are also adequate at moderate SNR. The worst throughput has 15 kHz SCS, with it having a longer symbol duration. It is most suitable for fixed or low-frequency channels. The most challenging mobile applications are best suited by 120 kHz SCS, whereas older or fixed installations are better supported by 15 kHz SCS.

#### Throughput vs modulation

To evaluate the modulation-dependent link performance in 5G NR, the throughput characteristics of QPSK, 16-QAM, 64-QAM, and 256-QAM were examined over an SNR range of − 10 to 20 dB.QPSK maintains reliable demodulation even under severe noise and fading conditions due to its large Euclidean symbol spacing and low constellation density, enabling superior throughput performance in low-SNR regions. In contrast, 256-QAM exhibits noticeably degraded performance in such conditions because of its reduced minimum distance between constellation points and high spectral efficiency requirements, resulting in increased symbol error probability unless the SNR is sufficiently high.

**Fig. 10:**
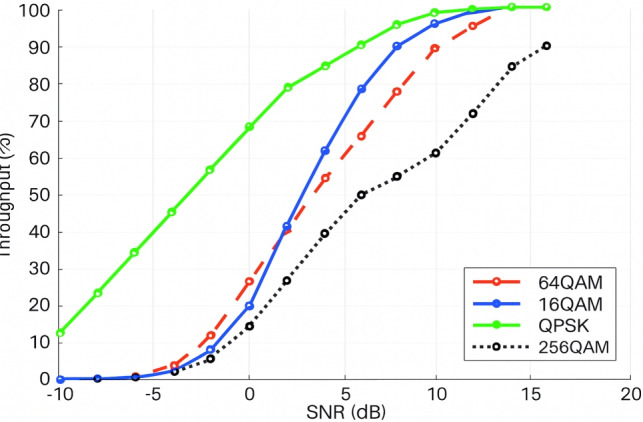
Evaluation of PDSCH BLER under Different Modulation Orders including QPSK, 16-QAM, 64-QAM, and 256-QAM in 5G NR.

The intermediate modulation formats, 16-QAM and 64-QAM, demonstrate transitional behavior with moderate performance in the mid-SNR region and show monotonic throughput improvement as SNR increases. In dense urban small-cell environments, where beamforming and frequency-selective fading predominate, these results emphasize the crucial role of Adaptive Modulation and Coding (AMC) in 5G NR physical-layer design. AMC enables the dynamic selection of an appropriate modulation order to maximize spectral efficiency while satisfying BLER constraints, as illustrated in Fig. 10.

#### BLER vs channel model

**Fig. 11:**
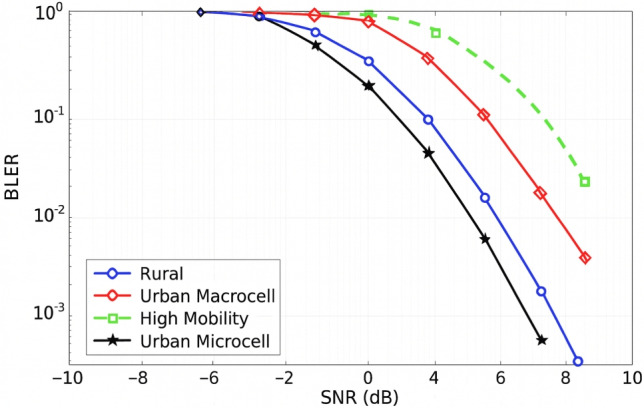
Downlink PDSCH BLER performance in 5G NR: macrocell deployments (15 kHz), high-mobility (60 kHz), rural (15 kHz), and urban (30 kHz) in 5G NR.

BLER Performance in Urban, Rural, and High-Mobility Scenarios: To assess the BLER performance in four representative propagation scenarios:Rural Macrocell (RMa), Urban Macrocell (UMa), Urban Microcell (UMi), and High-Speed Train (HST). Figure 11 was generated using standardized 3GPP TR 38.901 channel models. The simulations were conducted with a system bandwidth of 20 MHz and a carrier frequency of 3.5 GHz. The subcarrier spacing (SCS) configurations are 15 kHz for RMa and UMa, 30 kHz for UMi, and 60 kHz for the HST scenario were selected according to standard deployment guidelines.

Doppler frequencies ranging from approximately 10 Hz to 970 Hz were induced by mobility profiles of 3 km/h (UMi), 30 km/h (UMa), 120 km/h (RMa), and 300 km/h (HST), respectively. Channel delay spreads adhered to the relevant 3GPP specifications, with RMS values ranging from 45 ns to 300 ns depending on the propagation environment.

To isolate the intrinsic BLER characteristics without retransmission influence, 1000 transport blocks were transmitted using QPSK modulation in each scenario with HARQ disabled. A single-antenna (1$\times$1) configuration with perfect synchronization was assumed. The BLER curves were obtained by sweeping the signal-to-noise ratio (SNR) from − 10 dB to +10 dB.

###  Uplink PUCCH performance

####  BLER vs subcarrier spacing

**Fig. 12:**
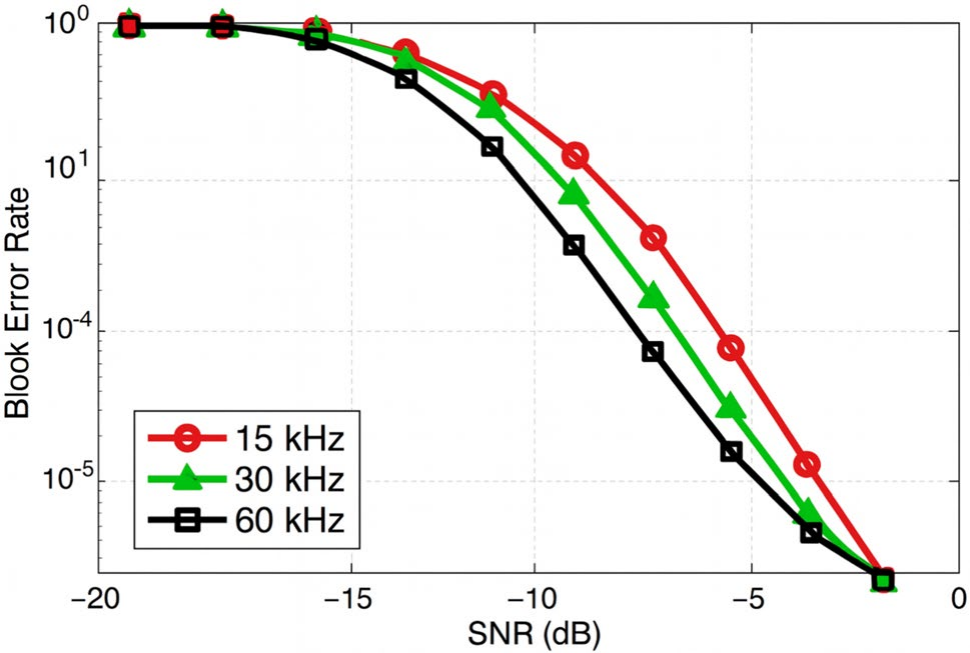
Assessment of PUCCH BLER in uplink control transmission with subcarrier spacings of 15 kHz, 30 kHz, and 60 kHz in 5G NR.

In the provided Fig. 12, BLER performance is compared across Subcarrier Spacings (SCS) of 15 kHz, 30 kHz, and 60 kHz over an SNR range from − 20 dB to 0 dB. The 60 kHz SCS demonstrates the best performance, exhibiting the lowest BLER throughout the entire SNR range. For example, at –10 dB SNR, the 60 kHz configuration achieves a BLER of approximately $10^{-2}$, while 30 kHz and 15 kHz configurations result in higher error rates of about $3 \times 10^{-2}$ and $5 \times 10^{-2}$, respectively. These results indicate that 60 kHz SCS offers greater robustness against fading and noise in challenging channel environments. The 30 kHz SCS provides moderate performance, whereas 15 kHz SCS demonstrates the poorest error resilience, with BLER exceeding $10^{-1}$ at low SNR values, rendering it unsuitable for reliability-critical applications. overall, the 60 kHz SCS is deemed optimal for high-mobility and high-reliability use cases in 5G NR systems, while 15 kHz remains better suited for legacy or wide-area coverage scenarios with relaxed reliability demands. In contrast to PUSCH, PUCCH offers more frequency variety and a higher DMRS density for bigger SCS values like 30 kHz and 60 kHz. It also includes brief control information. Higher SCS performs better in PUCCH transmissions because of these characteristics, which also improve robustness against phase noise and improve BLER performance for control signalling.

#### BLER vs frequency hopping

**Fig. 13:**
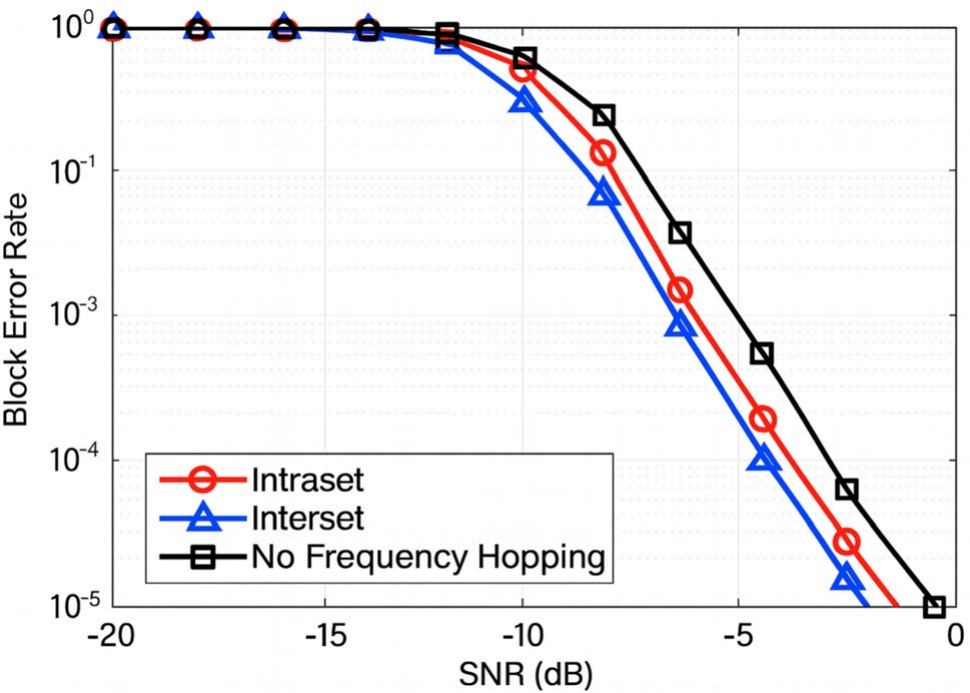
Interset hopping, intraset hopping, and no hopping over -20 dB to 0 dB are evaluated using PUCCH BLER in comparison to SNR in 5G NR.

The performance of BLER against SNR was evaluated for three frequency hopping schemes interset hopping, intraset hopping, and no hopping over an SNR range from − 20 to 0 dB, as shown in Fig. 13. Frequency hopping was identified as a critical mechanism in 5G NR, PUCCH for enhancing link reliability through frequency diversity. Among the tested schemes, interset frequency hopping yielded the lowest BLER, demonstrating superior resilience to frequency-selective fading and deep fades. Intraset hopping exhibited moderate reliability, while the no-hopping configuration resulted in the poorest performance, particularly under low-SNR conditions. For instance, at − 10 dB SNR, interset hopping achieved a BLER near $10^{-2}$, whereas significantly higher BLER values were recorded in the absence of hopping. Consequently, interset frequency hopping has been recognized as the most effective strategy for ensuring robust uplink control signaling in 5G NR systems.

#### Throughput vs varying frequency hopping

Throughput performance with respect to SNR has been evaluated for 5G NR PUCCH configurations, including interset frequency hopping, intraset frequency hopping, and the no-hopping scenario, under a 15 kHz Subcarrier Spacing (SCS) typical of sub-6 GHz systems. Frequency diversity was introduced through hopping mechanisms. Interset hopping enabled transmission across multiple frequency sets, thereby offering improved resistance against frequency-selective fading. In contrast, intraset hopping remained limited to a single frequency subset, providing only moderate performance improvement. Over the evaluated SNR range of –20 to 0 dB, interset hopping demonstrated the highest throughput, particularly between − 13 and − 8 dB SNR, where the benefits of frequency diversity were most pronounced. The no-hopping configuration exhibited the lowest throughput, attributed to its susceptibility to localized fading. Consequently, interset frequency hopping has been recognized as the most effective scheme for achieving optimal throughput and robust uplink control signaling in 5G NR systems.

#### Throughput vs varying channel model

**Fig. 14:**
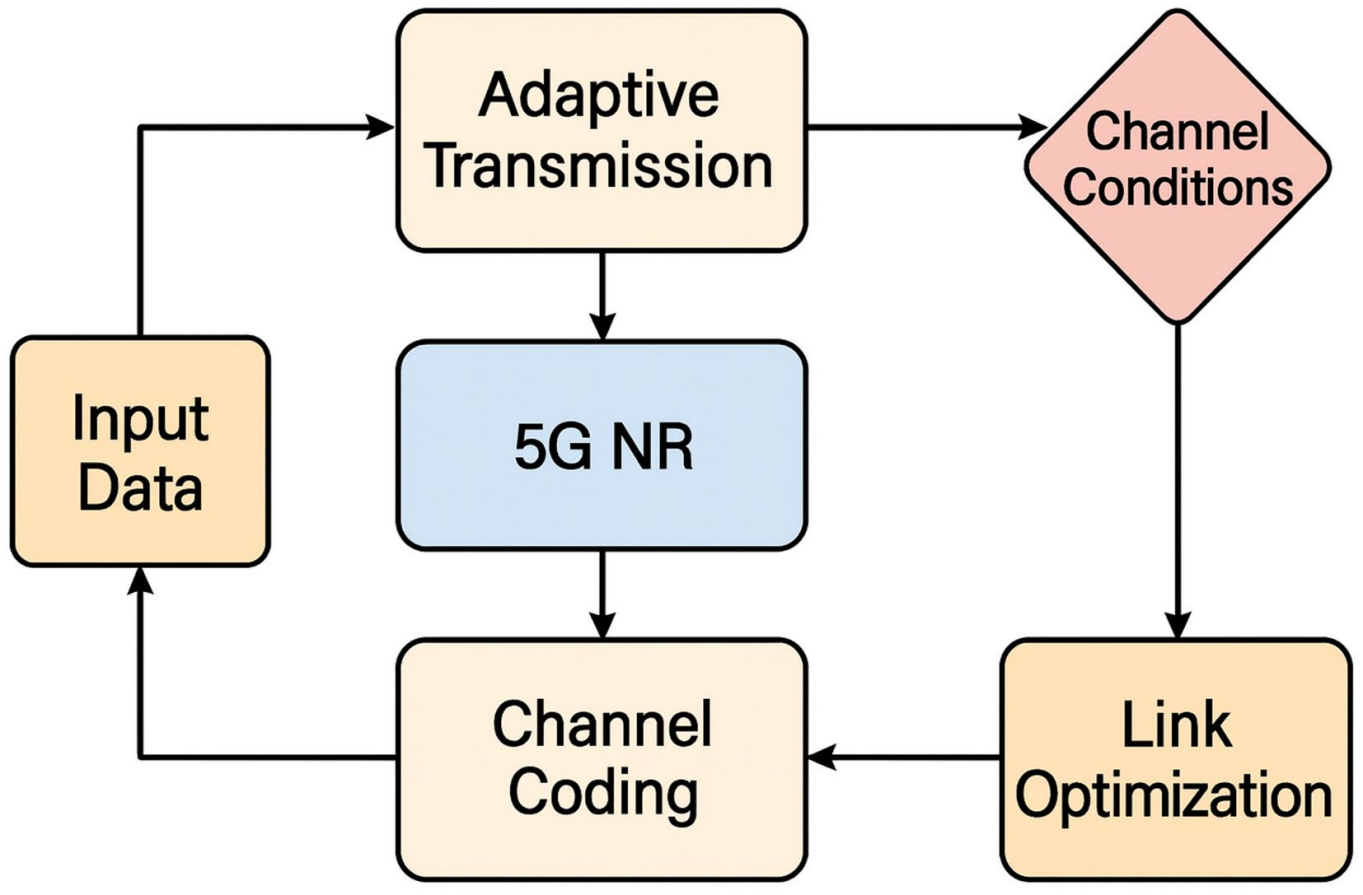
Proposed future research directions in 5G NR link-layer performance.

Throughput performance, measured in bits per transmission, has been evaluated as a function of SNR under strong, medium, and low multipath propagation conditions across an SNR range from –20 dB to 0 dB. Multipath propagation has been characterized by the presence of multiple delayed signal replicas caused by reflection and scattering. When appropriately leveraged through equalization and accurate channel estimation, these conditions have been shown to enhance robustness and system throughput, particularly in environments with strong multipath effects. Under such conditions, a peak throughput of 16 bits per transmission has been recorded at 0 dB SNR. Conversely, low multi-path scenarios, where frequency diversity is minimal, have resulted in the poorest throughput performance. For example, at –10 dB SNR, throughput under strong multi-path conditions reached approximately 11.5 bits, while low multi path yielded only 9.5  bits. These findings suggest that strong multi-path environments are more conducive to achieving optimal uplink control throughput in 5G NR systems, as shown in Fig. 14.

#### Additional results

Additional Results for Optimizing 5G NR Link Layer Parameters for eMBB and URLLC Applications under Dynamic Channel and Transmission Configurations as in Table 3.

**Table 3:** Summary of findings additional details.

Test scenario	Best case	Worst case
PUSCH BLER vs. Channel Model	Low Multipath	Very Strong Multipath
PUSCH BLER vs. Subcarrier Spacing	15 kHz	120 kHz
PUSCH Throughput vs. Channel Model	Very Strong Multipath	Low Multipath
PUSCH Throughput vs. Subcarrier Spacing	15 kHz	120 kHz
PUCCH BLER vs. Subcarrier Spacing	60 kHz	15 kHz
PUCCH BLER vs. Frequency Hopping	Interference Frequency Hopping	No Frequency Hopping
PUCCH Throughput vs. Frequency Hopping	Interference Frequency Hopping	No Frequency Hopping
PDSCH BLER vs. Channel Model	Strong Multipath	Low Multipath
PDSCH Throughput vs. Subcarrier Spacing	120 kHz	15 kHz
PDSCH Throughput vs. Modulation Scheme	256-QAM at High SNR	QPSK at High SNR
PDSCH BLER vs. Channel Model	Urban Microcell (30 kHz SCS)	Urban Macrocell (15 kHz SCS)

###  Summary of findings

The throughput performance of the PUCCH in 5G NR was evaluated under strong, medium, and weak multipath fading environments, across an SNR range of − 20 to 0 dB. Superior throughput was achieved in strong multipath conditions due to enhanced frequency diversity, which was effectively exploited through accurate channel estimation and equalization.Under strong multipath conditions, a maximum of 16 bits per transmission was recorded at 0 dB SNR,whereas weak multipath conditions scenarios yielded significantly lower throughput approximately, 9.5 bits per transmission at − 10 dB SNR due to insufficient multipath components for diversity gain. These results confirm that severe multipath fading conditions provide the optimal environment for achieving maximum PUCCH throughput, while weak multipath leads to degraded performance owing to Reduced robustness against channel impairments.

## Discussion

The findings highlight how crucial it is to adjust link settings adaptively in order to improve 5G NR performance under a variety of deployment scenarios. Because it maintains backward compatibility with LTE infrastructure and allows for wide-area coverage with good link quality in low-mobility situations, a 15 kHz subcarrier spacing is beneficial for rural and macro-cell environments. On the other hand, a 120 kHz subcarrier spacing is advantageous for mmWave deployments and extremely crowded, high-mobility urban environments because the shorter symbol duration enhances resilience to Doppler effects and reduces latency, two important factors that enable URLLC. Additionally, by offering frequency diversity and reducing frequency-selective fading, frequency hopping significantly increases the reliability of uplink control channels under severe fading and interference regimes typical of crowded metropolitan environments. All of these results suggest that in order to satisfy the various needs of modern 5G applications and deployment scenarios, subcarrier spacing and hopping methods should be dynamically adjusted to the propagation context.

## Conclusion and future work

The performance of important 5G NR link-layer operations, such as PDSCH, PUSCH, and PUCCH, under dynamic transmission parameters and standardised channel models was examined using a modelling framework based on MATLAB that complies with 3GPP. The results showed that modulation order, multipath fading, and subcarrier spacing have a significant impact on throughput and BLER. Higher subcarrier spacings, like 120 kHz, were shown to boost downlink throughput in high-frequency and high-mobility scenarios, while lower subcarrier spacings, like 15 kHz, were found to considerably improve uplink BLER performance in delay-spread situations. Moreover, QPSK offers reliable performance in situations with noise limitations, but higher-order modulation methods such as 256-QAM optimize throughput in advantageous SNR regimes. With the lowest BLER in fading conditions, PUCCH with interest frequency hopping demonstrated exceptional reliability. The study’s overall findings emphasise the need for adaptive link-layer configurations to strike a compromise between reliability and throughput, guaranteeing effective spectrum use. These findings offer important information for the design and optimization of 5G NR systems to satisfy the various needs of applications requiring URLLC and improved mobile broadband (eMBB).

It has been confirmed that multipath diversity greatly increases throughput, especially in situations with high fading when sophisticated channel coding and equalization techniques are used^39^. Moreover, significant gains in PUCCH reliability and spectrum efficiency were shown using frequency hopping techniques, particularly inter-set hopping^1^.

In summary, 5G NR link-layer performance optimization requires control signaling techniques, resilient channel coding, and adaptive transmission parameter selection^40–42^.

To confirm and expand on these findings, future research will concentrate on hardware-in-the-loop (HIL) testing and more investigation of link adaptation in massive MIMO environments and ultra-dense network deployments.

## Data Availability

The datasets used and/or analysed during the current study are available from the corresponding author on reasonable request.

## Appendix information

### Transport block formation and channel coding

If the size surpasses a threshold, $K_{\max }$, the gNB will partition the **Transport Block (TB)**, which is made up of *K* information bits, into smaller **Code Blocks (CBs)**. A codeword of length *N* is produced by encoding each CB using **Low-Density Parity-Check (LDPC)** as described in^7,17^. The formula for the code rate, as shown in Eq. (1) $R_c$, is

1 $$\begin{aligned} R_c \triangleq \frac{K}{N} \end{aligned}$$

Following encoding, **bit interleaving** and **rate matching** are used to modify the output to fit the physical resources at hand. Following the encoding process, bit interleaving and rate matching are applied to adapt the encoded output to the available physical resources^2,17^.

### Modulation and symbol mapping

The encoded bit stream is modulated using M-ary quadrature amplitude modulation (M-QAM). The equation gives the number of bits per symbol that corresponds to a modulation order as shown in Eq. (2) *M*^1,43^.

2 $$\begin{aligned} \eta _{\textrm{mod}} = \log _{2} M \end{aligned}$$

It is indicated that the modulation order *M* in 5G NR can be chosen from 4 (QPSK), 16, 64, and 256.The configured MIMO scheme determines how the complex-valued modulated signals, represented by the notation $\{ s_{1}, s_{2}, \dots , s_{N} \}$, are transferred to the transmission layers^2,17^.

### Layer mapping and precoding

The modulated symbols are first mapped to *L* transmission layers for **spatial multiplexing**, and then a matrix is used for *precoding*, $\textbf{F} \in \mathbb {C}^{N_t \times L}$, where $N_t$ is the number of transmit antennas.as shown in Eq. (3) The transmit signal vector is computed as^44^ :

3 $$\begin{aligned} \textbf{x} = \textbf{W} \cdot \textbf{s} \end{aligned}$$

*Precoding* aims to optimize **beam forming gain**, mitigate **inter-layer interference**, and adapt to **channel spatial properties**^45^.

### OFDM symbol generation and resource mapping

The pre-coded symbols are mapped onto the **time-frequency resource grid** using OFDM.as shown in Eq. (4) Given subcarrier spacing $\Delta f$, the duration of one OFDM symbol is:

4 $$\begin{aligned} T_{\textrm{sym}} = \frac{1}{\Delta f} \hspace{1cm} \end{aligned}$$

as shown in Eq. (5)^46^. The baseband signal is constructed via an inverse fast Fourier transform (IFFT):

5 $$\begin{aligned} x(t) = \sum _{n=0}^{N_{\textrm{SC}}-1} X[n] e^{j 2 \pi n \Delta f t} \hspace{1cm} \end{aligned}$$

where, $N_{\textrm{SC}}$ represents the number of subcarriers. A cyclic prefix is inserted before each symbol to mitigate inter-symbol interference^2^.

### DFT-spread OFDM and precoding

Discrete Fourier Transform-spread OFDM (DFT-S-OFDM) is supported for the Physical Up-link Shared Channel (PUSCH) in 5G NR to improve power efficiency. Before being mapped onto subcarriers, the input symbol stream is first converted to the frequency domain via a DFT operation as shown in Eq. (6).

6 $$\begin{aligned} X[k] = \sum _{n=0}^{N_{SC}-1} x[n] e^{-j \frac{2\pi nk}{N}} \end{aligned}$$

### Hybrid automatic repeat request (HARQ) mechanism

HARQ in 5G NR is implemented with **soft combining** at the receiver. Upon a decoding failure, retransmissions are triggered using different **Redundancy Versions (RVs)**. Let $\gamma _i$ represent the instantaneous SNR for the $i^{\text {th}}$ HARQ transmission. As shown in Eq. (7). The *effective SNR* after *n* retransmissions is

7 $$\begin{aligned} \gamma _{\text {eff}} = \sum _{i=1}^{n} \gamma _i \end{aligned}$$

Hybrid Automatic Repeat Request (HARQ), which uses soft combining at the receiver, is implemented in 5G NR. Different Redundancy Versions (RVs) are used to trigger retransmissions in the event of a decoding failure. For the $i^{\text {th}}$ HARQ transmission, let $\gamma _i$ represent the instantaneous signal-to-noise ratio (SNR). Following *n* retransmissions, the effective SNR is written as in Eq. (6)^47^. As shown in Eq. (8). The **probability of decoding failure** after *n* transmissions is given by

8 $$\begin{aligned} P_e(n) = \prod _{i=1}^{n} \left( 1 - P_S(i) \right) \end{aligned}$$

where $P_S(i)$ is denoted as the decoding success probability at the $i^{\text {th}}$ transmission attempt^2,24^. In the downlink, a maximum of 16 HARQ processes is permitted per User Equipment (UE), while up to 8 processes are allowed in the uplink. For each HARQ process, buffer resources, scheduling coordination, and timing alignment are required^4^.

### Link adaptation and scheduling

A key approach for throughput maximization in constantly changing channel circumstances is presented. The gNB chooses a modulation and coding scheme (MCS), based on the Channel Quality Indicator (CQI) feedback, in order to meet the target block error rate (BLER), $\varepsilon$, which is often set at $10\%$. as shown in Eq. (9). The feasible rate of data can be written as^36,47^.

9 $$\begin{aligned} R_{\text {ach}} = R_c \cdot \log _2(M) \cdot \frac{N_{\text {RB}} \times 12}{T_{\text {slot}}} \end{aligned}$$

As shown in Eq. (10) where $N_{\text {RB}}$ is the number of allocated resource blocks, and the slot duration $T_{\text {slot}}$ is defined by

10 $$\begin{aligned} T_{\text {slot}} = \frac{1}{2^n \cdot 1000} \end{aligned}$$

for $\Delta f = 2^n \cdot 15 \ \text {kHz}$. Scheduling is performed via the PDCCH, considering priority metrics such as **QoS Class Identifier (QCI)**, **Buffer Status Reports (BSR)**, and **channel conditions**^11^.

### Channel modeling and environmental effects

Sophisticated channel models are required to evaluate 5G NR performance realistically. Several models (such as CDL-A, CDL-B, CDL-C, and CDL-D) are described in the 3GPP TR 38.901 as shown in Eq. (11) specification to take into consideration the following features^12^ Path Loss is

11 $$\begin{aligned} PL(d) = 10 \cdot \alpha \cdot \log _{10}(d) + \beta + \chi _{\sigma } \end{aligned}$$

Doppler Spread:

12 $$\begin{aligned} f_D = \frac{v \cdot f_c}{c} \end{aligned}$$

Angle spread and delay spread affect spatial correlation and channel frequency as shown in Eq. (12) Urban macro (UMa), urban micro (UMi), rural macro (RMa), and interior environments are all reflected in the models, which are parameterized for sub-6 GHz and millimeterwave frequency ranges^48^.

### Frequency hopping, DMRS bundling, and PUCCH robustness

Interslot frequency hopping is used to improve spectrum diversity and reduce co-channel interference,as described by 3GPP-defined patterns. DMRS bundling, which aggregates several reference symbols to efficiently reduce estimate error variance, is used to improve channel estimation under low SNR situations.

Additionally, to improve the resilience of control signals for URLLC scenarios in difficult propagation conditions, frequency hopping in conjunction with repetition coding in PUCCH forms is employed.The model for the effective control channel SINR is as follows^1,49^.

13 $$\begin{aligned} \gamma _{\text {ctrl}}^{\text {eff}} = \frac{1}{LK} \sum _{l=1}^{L} \sum _{k=1}^{K} \gamma _{l,k} \end{aligned}$$

As shown in Eq. (13). Both simulation-based and hardware-in-the-loop testbeds have shown improved performance with these techniques^41,50^.

## Declarations

### Third party material

The authors declare that no third-party material has been used in the preparation of this manuscript.

## References

[1] 3rd Generation Partnership Project (3GPP). Nr; physical channels and modulation. Technical Specification TS 38.211 V17.6.0, 3GPP (2024). Release 17.

[2] Dahlman, E., Parkvall, S. & Sköld, J. *5G NR: The Next Generation Wireless Access Technology* (Academic Press, 2020).

[3] Sesia, S., Toufik, I. & Baker, M. *5G NR: Architecture Technology, Implementation* (Wiley, 2020).

[4] Lee, J. & Gil, J. An efficient HARQ mechanism for 5G new radio. *Sensors* **20**, 3972 (2020).32708924

[5] Kabalcı, Y. & Ali, M. Throughput analysis over 5g nr physical uplink shared channels. In *2nd Global Power. Energy and Communication Conference (GPECOM)* **345–349** (2020). 10.1109/GPECOM49333.2020.9247906 (2020).

[6] Yi, J., Kim, Y. & Ryu, H. Performance of uplink coverage enhancement schemes for 5G NR in 3gpp. In *2022 IEEE 95th Vehicular Technology Conference: (VTC2022-Spring)*, 1–5, 10.1109/VTC2022-Spring54318.2022.9860531 (2022).

[7] Kumar, R., Sinwar, D. & Singh, V. 5g new radio physical downlink shared channel throughput analysis with different numerology and modulation schemes. In *Soft Computing: Theories and Applications: Proceedings of SoCTA 2022*, 733–742 (Springer, 2023).

[8] Angelov, K., Sadinov, S. & Kogias, P. Modelling and study of the downlink physical layer in 5g nr mobile network. In *2022 International Conference on Communications, Information, Electronic and Energy Systems (CIEES)*, 1–4. 10.1109/CIEES55704.2022.9990892 (2022).

[9] Alqwider, W., Dahal, A. & Marojevic, V. Software radio with matlab toolbox for 5g nr waveform generation. In *2022 18th International Conference on Distributed Computing in Sensor Systems (DCOSS)*, 430–433. 10.1109/DCOSS54816.2022.00078 (2022).

[10] Salous, S. *et al.* Chapter 3 - iracon channel measurements and models. In *Inclusive Radio Communications for 5G and Beyond* (eds Oestges, C. & Quitin, F.) 49–105. 10.1016/B978-0-12-820581-5.00009-2 (Academic Press, 2021).

[11] Bug, S. & Jakoby, R. First results of modeling the mobile radio channel using theory of dynamics. *Frequenz* **57**, 226–231 (2003).

[12] 3rd Generation Partnership Project (3GPP). Study on channel model for frequencies from 0.5 to 100 GHz. Technical Report TR 38.901 V16.1.0, 3GPP (2019). Release 16.

[13] Li, N., Yir, H., Wang, B. & Zhu, J. Inter-slot frequency hopping with dmrs bundling for nr coverage enhancement. In *2023 26th International Symposium on Wireless Personal Multimedia Communications (WPMC)*, 1–5. 10.1109/WPMC59531.2023.10338964 (2023).

[14] Sesia, S., Toufik, I. & Baker, M. *5G NR: The Next Generation Wireless Access Technology* (Academic Press, 2016).

[15] Zaidi, A. *et al.* Waveform and numerology to support 5g services and requirements. *IEEE Commun. Mag.* **54**, 90–98. 10.1109/MCOM.2016.1600178CM (2016).

[16] MathWorks. *5G Toolbox: Simulate 5G New Radio Link-Level and System-Level Performance* (2024).

[17] 3rd Generation Partnership Project (3GPP). Nr; multiplexing and channel coding. Technical Specification TS 38.212 V17.6.0, 3GPP (2024). Release 17.

[18] Richardson, T. & Urbanke, R. *Modern Coding Theory* (Cambridge University Press, 2008).

[19] Santos, R., Castanheira, D., Silva, A. & Gameiro, A. Pipelined multi-user IR-HARQ scheme for improved latency performance in URLLC. *IEEE Access* **12**, 33473–33485 (2024).

[20] Mahatme, P. M. R., Kale, P. S. & Prasad, N. R. Performance analysis of physical uplink shared channel (pusch) in 5g nr. In *Proceedings of the IEEE International Conference on Electronics, Computing and Communication Technologies (CONECCT)*, 1–6, 10.1109/CONNECT52877.2021.9500247 (2021).

[21] Wang, H.*et al.* The -- fading distribution. *IEEE Antennas Wirel. Propag. Lett.* **23**, 4398–4402 (2024).

[22] Liu, J. *et al.* Double -- distribution: Modeling and performance evaluation in non-homogeneous environments. *IEEE Wirel. Commun. Lett.* **14**, 3378–3382 (2025).

[23] Kibria, M. G. *et al.* A tutorial on 5g nr downlink: Features, performance, and open challenges. *IEEE Access* **8**, 166248–166284. 10.1109/ACCESS.2020.3022537 (2020).

[24] 3rd Generation Partnership Project (3GPP). Nr; physical layer procedures for data. Technical Specification TS 38.214 V17.6.0, 3GPP (2024). Release 17.

[25] Rumney, M. *5G NR: The Next Generation Wireless Access Technology* 2 edn (Wiley, 2020).

[26] Parkvall, S., Dahlman, E. & Furuskar, A. Nr: The new 5g radio access technology. *IEEE Commun. Stand. Mag.* **1**, 24–30 (2017).

[27] Lauridsen, M. *et al.* An empirical evaluation of HARQ in 5g nr. *IEEE Access* **8**, 45069–45080 (2020).

[28] Sormunen, L. *et al.* Simulative comparison of dvb-s2x/rcs2 and 3gpp 5g nr ntn technologies in a geostationary satellite scenario. In *2025 12th Advanced Satellite Multimedia Systems Conference and the 18th Signal Processing for Space Communications Workshop (ASMS/SPSC)*, 1–8 (IEEE, 2025).

[29] Rahmani, M. *et al.* Error rate performance analysis of the 5g nr physical uplink shared channels for cell-free systems. *Authorea Preprints* (2024).

[30] Ray, J. K., Bera, R., Sil, S. & Alfred, Q. M. Analysis of BLER and throughput during the coexistence of two 5G NR. In *Doctoral Symposium on Human Centered Computing*, 289–301 (Springer, 2023).

[31] Jyoti, A. & Kumar, D. Investigating resource allocation techniques and key performance indicators (KPIs) for 5G new radio networks: A review. *Int. J. Comput. Netw. Appl.* **10**, 422–442 (2023).

[32] Lapin, V. & Veyler, A. Error rate performance analysis of the 5g new radio physical uplink control channel. In *2022 International Conference on Modern Network Technologies (MoNeTec)*, 1–7 (IEEE, 2022).

[33] Varatharaajan, S., Grossmann, M. & Del Galdo, G. 5G new radio physical downlink control channel reliability enhancements for multiple transmission-reception-point communications. *IEEE Access* **10**, 97394–97407 (2022).

[34] Hao, P., Han, X., Xia, S., Ren, M. & Deng, Y. Performance evaluation of 5g ultra-reliable and low latency communications. In *2020 International Wireless Communications and Mobile Computing (IWCMC)*, 1047–1052 (IEEE, 2020).

[35] Jiang, T., Li, Y. & Zhang, H. LDPC codes for 5G new radio: Design considerations and performance evaluation. *IEEE Wirel. Commun.* **27**, 70–77. 10.1109/MWC.001.1900201 (2020).

[36] Zaidi, A. *et al.* Waveform and numerology to support 5G services and requirements. *IEEE Commun. Mag.* **54**, 90–98. 10.1109/MCOM.2016.1600332CM (2016).

[37] *MathWorks. Nr pusch throughput (5g toolbox)*, accessed 26 August 2025; https://in.mathworks.com/help/5g/ug/nr-pusch-throughput.html.

[38] MathWorks. Nr pucch block error rate – matlab & simulink, accessed 25 August 2025; https://in.mathworks.com/help/5g/ug/nr-pucch-block-error-rate.html (2025).

[39] Marzetta, T. L., Larsson, E. G., Yang, H. & Ngo, H. Q. *Fundamentals of Massive MIMO* (Cambridge University Press, 2016).

[40] Anonymous. Genetic algorithm optimization of the link layer for throughput in 5g nr. *J. Mobile Multimed.* (2025). (Directly aligns with your GA-based link-layer tuning.).

[41] Ji, H. *et al.* Ultra-reliable and low-latency communications in 5g downlink: Physical layer aspects. *IEEE Wirel. Commun.* **25**, 124–130 (2018).

[42] Björnson, E., Hoydis, J. & Sanguinetti, L. Massive MIMO networks: Spectral, energy, and hardware efficiency. *Found. Trends Signal Process.* **11**, 154–655 (2017).

[43] Proakis, J. G. & Salehi, M. *Digital Communications* 5th edn (McGraw-Hill, 2007).

[44] Tse, D. & Viswanath, P. *Fundamentals of Wireless Communication* (Cambridge University Press, 2005).

[45] Marzetta, T. L., Larsson, E. G., Yang, H. & Ngo, H. Q. *Fundamentals of Massive MIMO* (Cambridge University Press, 2016).

[46] Anonymous. Flexible numerology in 5G NR: Interference quantification and proper selection depending on the scenario. *Mobile Inf. Syst.* (2021).

[47] Sesia, S., Toufik, I. & Baker, M. *5G NR: The Next Generation Wireless Access Technology*, 2nd edn (Academic Press, 2021).

[48] Sun, S., Rappaport, T. S., Rangan, S. *et al.* Propagation path loss models for 5g urban micro- and macro-cellular scenarios. *IEEE Vehicular Technology Conference (VTC Spring)* 1–6 (2016).

[49] 3rd generation partnership project; technical specification group radio access network; nr; physical layer procedures for control (release 15) (2018). V15.2.0.

[50] Anand, A. *et al.* Design of pucch for 5g nr and performance evaluation. In *IEEE International Conference on Communications Workshops (ICC Workshops)*, 1–6 (2018).

